# Prognostic value of heart rate deceleration capacity for functional outcomes in acute ischemic stroke: a prospective study

**DOI:** 10.3389/fendo.2025.1601346

**Published:** 2025-05-13

**Authors:** Huizhong Zhou, Jiaqi Zhong, Changman Deng, Xingde Wang, Yanhong Xu, Jiajun Yang

**Affiliations:** ^1^ Department of Neurology, Shanghai Sixth People’s Hospital Affiliated to Shanghai Jiao Tong University School of Medicine, Shanghai, China; ^2^ Shanghai Neurological Rare Disease Biobank and Precision Diagnostic Technical Service Platform, Shanghai, China; ^3^ Department of Cardiology, Shanghai Sixth People’s Hospital Affiliated to Shanghai Jiao Tong University School of Medicine, Shanghai, China

**Keywords:** acute ischemic stroke, autonomic dysfunction, deceleration capacity, heart rate variability, prognosis

## Abstract

**Purpose:**

Acute ischemic stroke (AIS) is a leading cause of disability and mortality, with poor functional outcomes often linked to autonomic dysfunction. Deceleration capacity (DC), a marker of vagal activity, has been shown to predict cardiovascular outcomes, but its prognostic value in AIS remains underexplored. This study investigates the role of DC in predicting stroke recovery at 2 weeks and 3 months post-stroke.

**Patients and methods:**

This prospective study included 423 AIS patients treated at a single center between January 2022 and December 2023. Cardiac autonomic function was assessed using DC and heart rate variability (HRV) parameters (SDNN, SDANN, RMSSD), derived from 24-hour Holter ECG monitoring. Patients were categorized into two groups based on DC: DC > 4.5 ms and DC ≤ 4.5 ms. Functional outcomes were measured using the modified Rankin Scale (mRS) at 2 weeks and 3 months post-stroke. Logistic regression models and Restricted Cubic Splines (RCS) were used to analyze the relationship between DC and stroke outcomes.

**Results:**

Patients with lower DC (≤4.5ms) had significantly worse functional outcomes, as indicated by higher mRS scores at both 2 weeks and 3 months. The DC ≤ 4.5 ms group also had a higher prevalence of comorbidities such as diabetes and hypertension. RCS analysis revealed a non-linear relationship between DC and stroke outcomes, with a significant threshold at DC = 4.5 ms. The 3-month outcome model, including age and DC, demonstrated strong predictive ability with an AUC of 0.744.

**Conclusions:**

This study highlights the importance of DC as a prognostic marker for short-term stroke recovery. Lower DC values are associated with worse outcomes, suggesting that DC may serve as an early predictor of stroke prognosis. Future research should focus on validating these findings in larger, multicenter cohorts and exploring interventions targeting autonomic dysfunction to improve stroke recovery.

## 1 Introduction

Acute ischemic stroke (AIS) remains a leading cause of disability and mortality worldwide (1). From 1990 to 2019, the global death toll from ischemic stroke (IS) increased from 2.04 million to 3.29 million, and it is projected to reach 4.90 million by 2030 (2). The prognosis of patients with AIS is influenced by factors such as stroke severity, timely interventions, and the presence of comorbidities (3). Therefore, the early prediction and intervention following AIS are of paramount significance to public health. However, a reliable indicator for predicting stroke prognosis remains lacking, posing a challenge for early assessment and treatment.

Autonomic nervous system (ANS) dysfunction is commonly observed in AIS patients and is associated with poor clinical outcomes (4). Cardiac autonomic function, in particular, has emerged as a critical factor in evaluating stroke severity and predicting stroke outcomes (5, 6). Traditional methods, such as heart rate variability (HRV), baroreflex sensitivity (BRS), have been widely used in this field (7, 8). However, these techniques have limitations in distinguishing between sympathetic and parasympathetic (vagal) nerve activities (5), and have not been well applied in clinical practice. Therefore, there is a growing need for more precise tools to evaluate autonomic function in stroke patients.

Phase-rectified signal averaging (PRSA) is a novel technique developed to assess cardiac autonomic function (9, 10). Unlike traditional HRV analysis, PRSA can quantitatively distinguish between vagal and sympathetic nerve activity through two key parameters: deceleration capacity (DC) and acceleration capacity (AC) (11).

DC, as a marker of vagal tone, has been shown to be a reliable predictor of mortality and outcomes in cardiovascular diseases. Research indicates that higher DC values are associated with a lower risk of mortality in conditions like myocardial infarction, heart failure and aging (10, 12, 13). Despite its established potential, the application of PRSA in stroke patients remains largely unexplored.

In this study, we conducted a single-center, prospective investigation to explore the potential value of autonomic function in predicting the prognosis of AIS. We chose DC as the main indicator of autonomic function, and found that DC is an independent predictive factor of three-month prognosis of AIS, though not of two-week prognosis.

## 2 Material and methods

### 2.1 Study design

A prospective study was conducted to explore the predictive value of DC in AIS patients at Shanghai Sixth People’s Hospital Lingang Branch from January 2022 to December 2023. Ethical approval for the study was obtained [Approval No. 2024-KY-001(K)], and informed consent was provided by all participants or their representatives.

### 2.2 Data collection

#### 2.2.1 Population and clinical data

Comprehensive inclusion and exclusion criteria were established for patient selection. Patients were eligible if they met all of the following criteria: diagnosis of AIS according to the 2019 Chinese Guidelines for AIS Diagnosis and Treatment (14), age of 18 years or older, admission within 24 hours of symptom onset, ability to undergo 24-hour Holter ECG monitoring, and provision of written informed consent. We excluded patients with any of the following conditions: a history of intracranial tumors; severe cardiac arrhythmias, including atrial fibrillation, frequent premature beats, or second-degree or higher atrioventricular block; concurrent malignancy or severe hepatic or renal dysfunction; diseases affecting autonomic nervous system function, such as hyperthyroidism or hypothyroidism; severe psychiatric disorders that could affect clinical assessment; infection within two weeks before stroke onset; or the use of medications that could influence autonomic nervous system function, including α/β-adrenergic receptor blockers or agonists, and corticosteroids. Of note, patients with cardioembolic stroke due to patent foramen ovale (PFO) were included in the study despite the exclusion of those with severe arrhythmias, such as atrial fibrillation.

For all eligible patients, comprehensive demographic and clinical data were collected from the hospital information system. These data included demographics information (age, sex, height, weight, Body Mass Index), medical history (hypertension, diabetes, smoking, alcohol consumption), clinical characteristics (admission NIHSS score, blood pressure, thrombolysis/thrombectomy treatment), laboratory data (complete blood count, blood glucose, HbA1c, lipid profile, uric acid), and imaging findings from brain MRI/CT and MRA/CTA scans. Etiological classification was performed according to the TOAST (Trial of Org 10172 in Acute Stroke Treatment) criteria (15).

#### 2.2.2 Autonomic function assessment

All participants underwent 24-hour Holter ECG monitoring within 48 hours of admission. The monitoring period was standardized from 8:00 AM to 8:00 AM the following day. ECG data were analyzed using Cardioscan-12 software. PRSA technique which was meticulously described in Bauer’s study was used to analyze the acceleration capacity and deceleration capacity of heart (10). Simply, PRSA is a signal processing technique that analyzes RR intervals through a five-step algorithm, identifying anchor points, selecting data segments, aligning and averaging signals, and quantifying heart rate variations using Haar wavelet analysis. Two types of autonomic parameters were assessed: First, DC, the primary parameter of interest, which specifically reflects cardiac vagal modulation. Based on previous studies and clinical relevance (10), DC values were dichotomized into two groups: DC > 4.5 ms and DC ≤ 4.5 ms for analysis. AC, which is thought to reflect the sympathetic function, was calculated using the same method as for DC. Additionally, traditional HRV parameters were also analyzed, including Standard Deviation of Normal to Normal Intervals (SDNN), which reflects overall autonomic activity, Standard Deviation of the Averages of Normal to Normal Intervals (SDANN), which reflects long-term variability in heart rate, and Root Mean Square of Successive Differences of Normal to Normal Intervals (RMSSD) which reflects vagal tone (16, 17). The selection of a 4.5 ms threshold for DC was based on multiple lines of evidence. Early studies demonstrated that a DC below 4.5 ms is closely associated with increased mortality risk in myocardial infarction patients, effectively distinguishing individuals with impaired vagal modulation (10). In addition, subsequent research in heart failure, drug-induced cardiotoxicity, and non-ischemic dilated cardiomyopathy (12, 13) has validated this threshold as a reliable marker to differentiate patients with normal from those with significantly compromised autonomic function. Moreover, receiver operating characteristic (ROC) analyses in these studies consistently showed that 4.5 ms represents an optimal cut-off, balancing sensitivity and specificity for risk stratification. Given the similar patterns of autonomic dysfunction observed in acute ischemic stroke patients, adopting the 4.5 ms threshold in our study is both scientifically justified and clinically relevant, as it facilitates early identification of patients at high risk of unfavorable outcomes.

### 2.3 Outcome assessment

The primary outcome was functional status assessed by the modified Rankin Scale (mRS) at both two weeks and three months after stroke. The mRS evaluations were conducted by trained neurologists who were blinded to the patients’ autonomic function. For patients unable to attend follow-up visits, assessments were conducted through telephone interviews with either patients or their caregivers. The mRS scores were dichotomized into two categories: favorable (0-2) and unfavorable (3-6) outcomes (18).

### 2.4 Statistical analysis

All statistical analyses were conducted using R software (version 4.2.2). Baseline characteristics and clinical data were summarized as mean ± standard deviation (SD) or median (interquartile range, IQR) for continuous variables, depending on their distribution, and as frequencies (percentages) for categorical variables. Normality was assessed using the Shapiro-Wilk test, and homogeneity of variance was examined using Levene’s test. Differences between groups (DC > 4.5 and DC ≤ 4.5) were compared using independent t-test for normally distributed variables, Mann-Whitney U test for non-normally distributed variables, and chi-square or Fisher’s exact test for categorical variables.

Correlation analyses were performed to evaluate associations between potential variables. Multicollinearity among variables was assessed using variance inflation factors (VIF), and variables with VIF > 5 were excluded from multivariate models. Logistic regression analysis was conducted to identify independent predictors of unfavorable outcomes (mRS 3–6) at both two weeks and three months post-stroke. Covariates included age, Body Mass Index (BMI), diabetes, hypertension, DC, SDNN, and RMSSD. Adjusted odds ratios (ORs) with 95% confidence intervals (CIs) were calculated.

Restricted cubic spline (RCS) models (19) were used to explore non-linear relationships between DC and mRS outcomes, identifying trends and thresholds around the clinically relevant cut-off of DC = 4.5ms. The RCS model was constructed with 3 knots placed at the 10th, 50th, and 90th percentiles of the DC distribution (corresponding to values of 3.05, 5.88, and 9.00 ms, respectively), providing a balance between model flexibility and stability. Model performance was evaluated using receiver operating characteristic (ROC) curve analysis, with the area under the curve (AUC) used to evaluate predictive accuracy. Statistical significance was set at P < 0.05 (two-tailed).

In the present study, the mRS scores were dichotomized into favorable (0–2) and unfavorable (3–6) outcomes. This dichotomization was performed because mRS scores are inherently ordinal and typically exhibit a non-normal, skewed distribution in stroke populations. Logistic regression is a robust method for modeling binary outcomes and provides easily interpretable odds ratios that quantify the association between DC and functional recovery. Additionally, logistic regression allows for adjustment of multiple confounding variables, ensuring a stable estimation of the effect size even when the underlying outcome distribution deviates from normality. This methodological approach is consistent with prior studies in the field and is well-suited for the prognostic analysis of AIS outcomes.

## 3 Results

### 3.1 Baseline characteristics and outcomes of AIS patients

**Figure 1:**
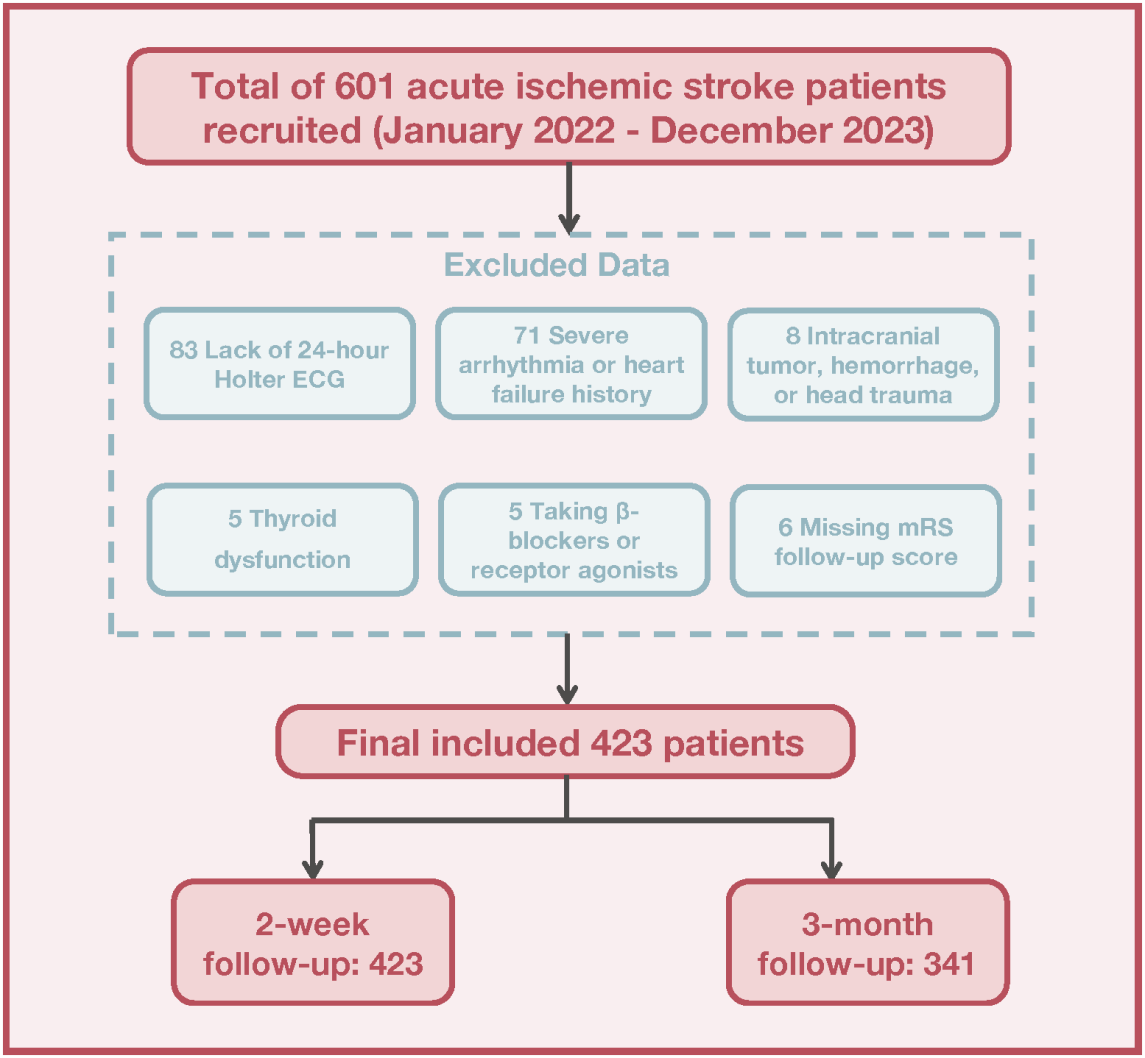
Flowchart depicting the recruitment and selection process of acute ischemic stroke patients.

Between January 2022 and December 2023, 601 patients with AIS were initially recruited. After exclusions for medical and procedural reasons, 423 patients remained for analysis. These patients were assessed using the mRS at both 2 weeks and 3 months, with 341 completing the 3-month follow-up (**Figure 1**). Significant differences were observed in the baseline characteristics between the DC > 4.5 and DC ≤ 4.5 groups. The DC ≤ 4.5 group was older (69 vs. 59 years, P < 0.001) and had a higher prevalence of diabetes (48.5% vs. 30.4%, P = 0.001) and hypertension (81.2% vs. 69.6%, P = 0.031) compared to the DC> 4.5 group. Other cardiac autonomic indicators, such as SDNN, SDANN, and rMSSD, were significantly lower in the DC≤ 4.5 group (P < 0.001 for all). Furthermore, the DC ≤ 4.5 group demonstrated worse functional outcomes on the mRS at both 2 weeks and 3 months after stroke (P < 0.001). Differences in stroke subgroups and vessel narrowing were also observed, with a higher proportion of small-artery occlusion and more severe vessel narrowing in the DC ≤ 4.5 group (P = 0.016 and P = 0.039, respectively). These findings indicate that reduced deceleration capacity is associated with older age, more comorbidities, and worse short-term outcomes, as summarized in **Table 1**.

**Table 1:** Baseline characteristics of the study population.

Variable	DC>4.5 (n=322)	DC ≤ 4.5 (n=101)	P Value
**Age (mean (SD))**	58.85 (11.79)	68.89 (12.27)	**<0.001**
Female (%)	84 (26.1)	32 (31.7)	0.331
**BMI (median [IQR])**	25.10 [23.38, 26.57]	24.35 [22.05, 25.71]	**0.036**
**Diabetes (%)**	98 (30.4)	49 (48.5)	**0.001**
**Hypertension (%)**	224 (69.6)	82 (81.2)	**0.031**
Coronary Heart Disease (%)	9 (2.8)	2 (2.0)	0.928
Previous Stroke History (%)	40 (12.4)	17 (16.8)	0.334
Smoking History (%)	89 (27.6)	18 (17.8)	0.064
Alcohol History (%)	48 (14.9)	8 (7.9)	0.101
mRS_2 weeks (n=423)			
**0-2 (%)**	237 (73.6)	53 (52.5)	**<0.001**
**3-6 (%)**	85 (26.4)	48 (47.5)	
mRS_3 months (%) (n=341)			
**0-2 (%)**	225 (69.9)	38 (37.6)	**<0.001**
**3-6 (%)**	37 (14.1)	41 (51.9)	
NIHSS at Admission (median [IQR])	2.00 [1.00, 4.00]	3.00 [1.00, 4.00]	0.087
Thrombolysis/thrombectomy (%)	55 (17.1)	17 (16.8)	1
TOAST_Subgroup (%)			
LAA	103 (32.0)	48 (47.5)	**0.016**
CE	14 (4.3)	0 (0.0)	
SAO	179 (55.6)	48 (47.5)	
SOE	22 (6.8)	5 (5.0)	
SUE	4 (1.2)	0 (0.0)	
Vessel_narrowing (%)			
None	150 (48.7)	39 (42.4)	**0.039**
Mild	43 (14.0)	20 (21.7)	
Moderate	28 (9.1)	4 (4.3)	
Severe	32 (10.4)	17 (18.5)	
Occlusion	55 (17.9)	12 (13.0)	
**|AC| (median [IQR])**	7.24 [5.80, 8.76]	3.39 [2.71, 3.95]	**<0.001**
**SDNN (median [IQR])**	109.00 [91.25, 131.00]	70.00 [57.00, 89.00]	**<0.001**
**SDANN (median [IQR])**	88.00 [70.00, 106.00]	60.00 [45.00, 77.00]	**<0.001**
**rMSSD (median [IQR])**	32.00 [23.00, 51.75]	22.00 [13.00, 36.00]	**<0.001**

LAA, Large-artery atherosclerosis; CE, Cardioembolism; SAO, Small-artery occlusion Lacunar; SOE, Other determined etiology; SUE, Undetermined etiology; BMI, Body Mass Index; NIHSS, National Institutes of Health Stroke Scale; DC, deceleration capacity; AC, acceleration capacity; SDNN, Standard Deviation of Normal to Normal Intervals; SDANN, Standard Deviation of the Averages of Normal to Normal Intervals; rMSSD, Root Mean Square of Successive Differences of Normal to Normal Intervals; mRS, Modified Rankin Scale.

Bold values indicate statistically significant differences (P < 0.05) between the DC > 4.5 ms and DC ≤ 4.5 ms groups.

### 3.2 Correlations between clinical parameters and variable selection

The correlation analysis presented in **Supplementary 1** revealed two pairs of strongly correlated autonomic parameters. An exceedingly strong negative correlation was observed between AC and DC (r = -0.96), while SDNN and SDANN showed significant positive correlation (r = 0.86). These pronounced correlations raise concerns about multicollinearity, which could potentially compromise the stability and accuracy of predictive models. To mitigate this issue, DC and SDNN were selected due to their strong representation of autonomic function. Additionally, clinical parameters, including age, BMI, diabetes, and hypertension, and the HRV metric rMSSD, were incorporated into the analysis.

### 3.3 Predictive modeling for stroke outcomes

Logistic regression models were developed to predict stroke outcomes at both 2 weeks and 3 months post-stroke, incorporating multiple variables, including age, BMI, diabetes, hypertension, DC, and HRV parameters (SDNN and rMSSD). mRS scores were dichotomized into favorable (mRS 0-2) and unfavorable (mRS 3-6) outcomes. At 2 weeks, age (OR = 1.03, 95% CI: 1.00-1.05, P = 0.044) and hypertension (OR = 1.92, 95% CI: 1.05-3.62, P = 0.037) emerged as significant predictors of unfavorable outcomes, while other variables including BMI (P = 0.885), diabetes (P = 0.230), DC (P = 0.106), SDNN (P = 0.650), and rMSSD (P = 0.690) showed no significant predictive value. The 3-month analysis revealed that age (OR = 1.05, 95% CI: 1.02-1.09, P = 0.004) and reduced DC (OR = 3.36, 95% CI: 1.51-7.51, P = 0.003) were robust predictors of poor functional outcomes, while BMI (P = 0.875), diabetes (P = 0.223), hypertension (P = 0.396), SDNN (P = 0.306), and rMSSD (P = 0.571) did not show significant associations.

**Figure 2:**
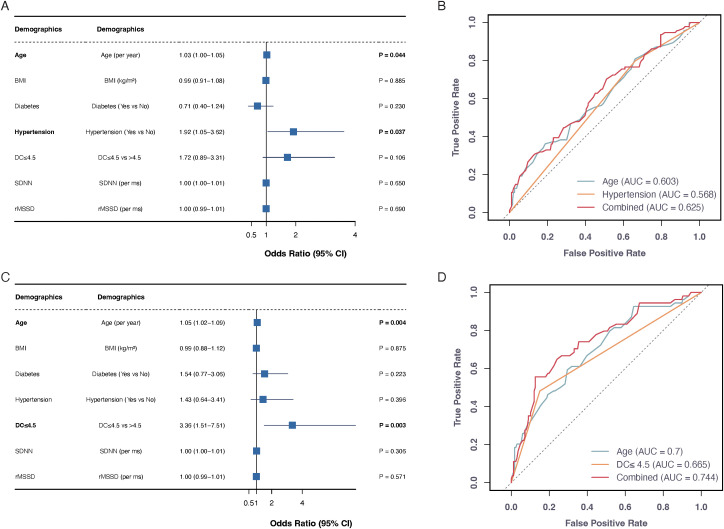
Predictive models for stroke functional outcomes at 2 weeks and 3 months. Forest plots **(A, C)** show odds ratios (95% CI) for unfavorable outcomes (mRS 3-6) at 2 weeks and 3 months post-stroke, respectively. ROC curves **(B, D)** display predictive performance of individual and combined factors. The age+hypertension combination achieved highest AUC (0.625) at 2 weeks, while age+DC combination showed best performance (AUC=0.744) at 3 months.

ROC curve analysis was performed to assess the discriminative capability of these predictive models (**Figure 2**). For 2-week outcomes, individual predictors showed modest performance (age: AUC = 0.603; hypertension: AUC = 0.568), with the combined model achieving an AUC of 0.625. The 3-month model demonstrated superior discriminative ability, with age alone achieving an AUC of 0.700, DC showing an AUC of 0.665, and their combination yielding the highest predictive performance (AUC = 0.744).

### 3.4 Non-linear relationship between DC and stroke outcomes

**Figure 3:**
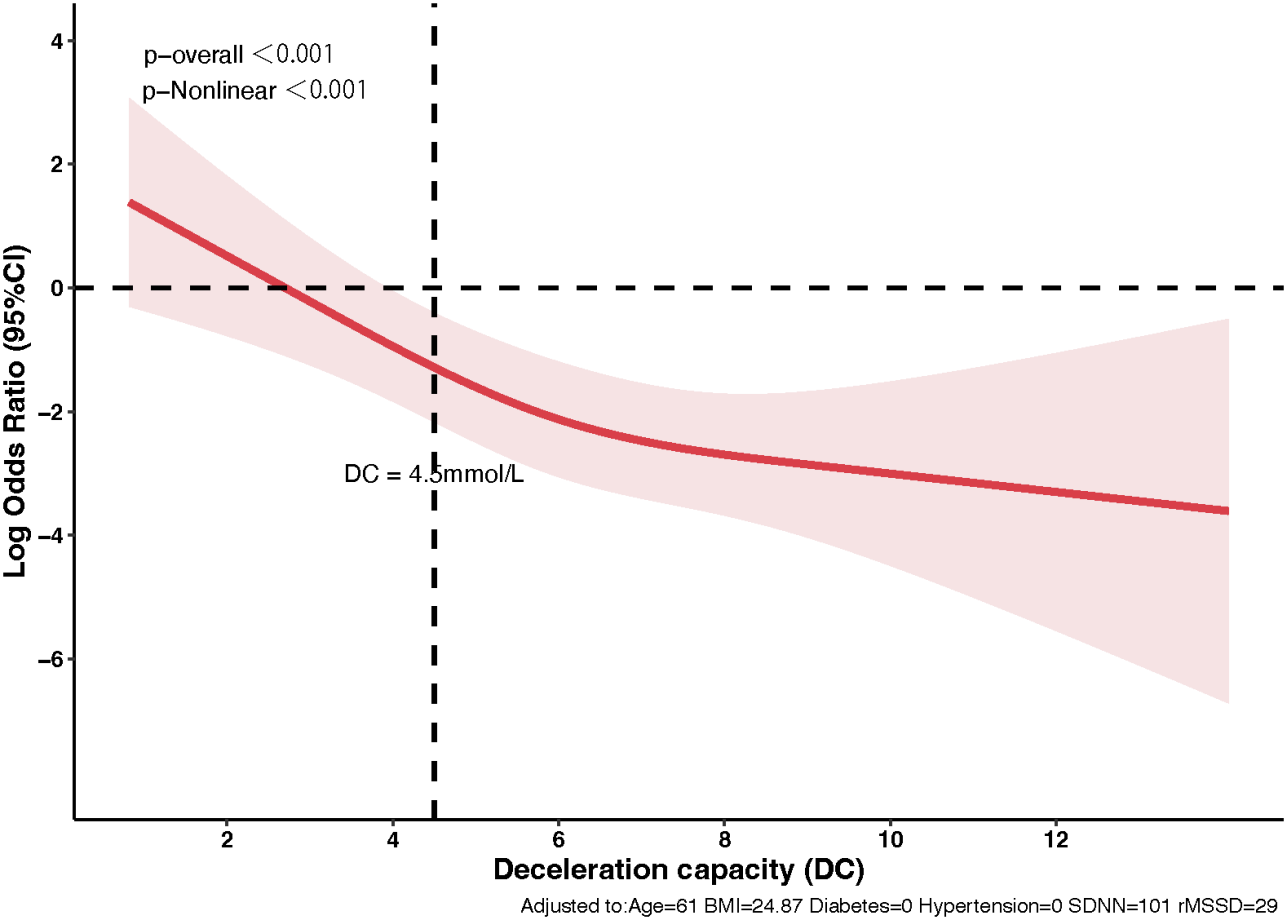
Non-linear dynamics of deceleration capacity in predicting stroke outcomes. The relationship between DC and 3-month stroke outcomes using Restricted Cubic Splines. The red line indicates odds ratios for unfavorable outcomes (mRS scores 3-6) with changes in DC, highlighting a significant decrease in risk beyond a DC of 4.5 ms. The shaded area represents the 95% confidence interval.

The relationship between DC and 3-month stroke outcomes, analyzed using logistic regression with RCS illustrated a significant non-linear effect (**Figure 3**). The analysis indicated that DC values below 4.5 ms were associated with substantially higher odds of unfavorable outcomes (mRS scores of 3-6). The RCS model identified a notable inflection point at the DC threshold of 4.5 ms, beyond which the relationship between DC and unfavorable outcomes became more gradual. This non-linear pattern suggests that while improvements in DC values above 4.5 ms continue to be associated with better outcomes, the most dramatic changes in risk occur within the lower range of DC values.

### 3.4 Functional outcomes by DC levels at 3 months

**Figure 4:**
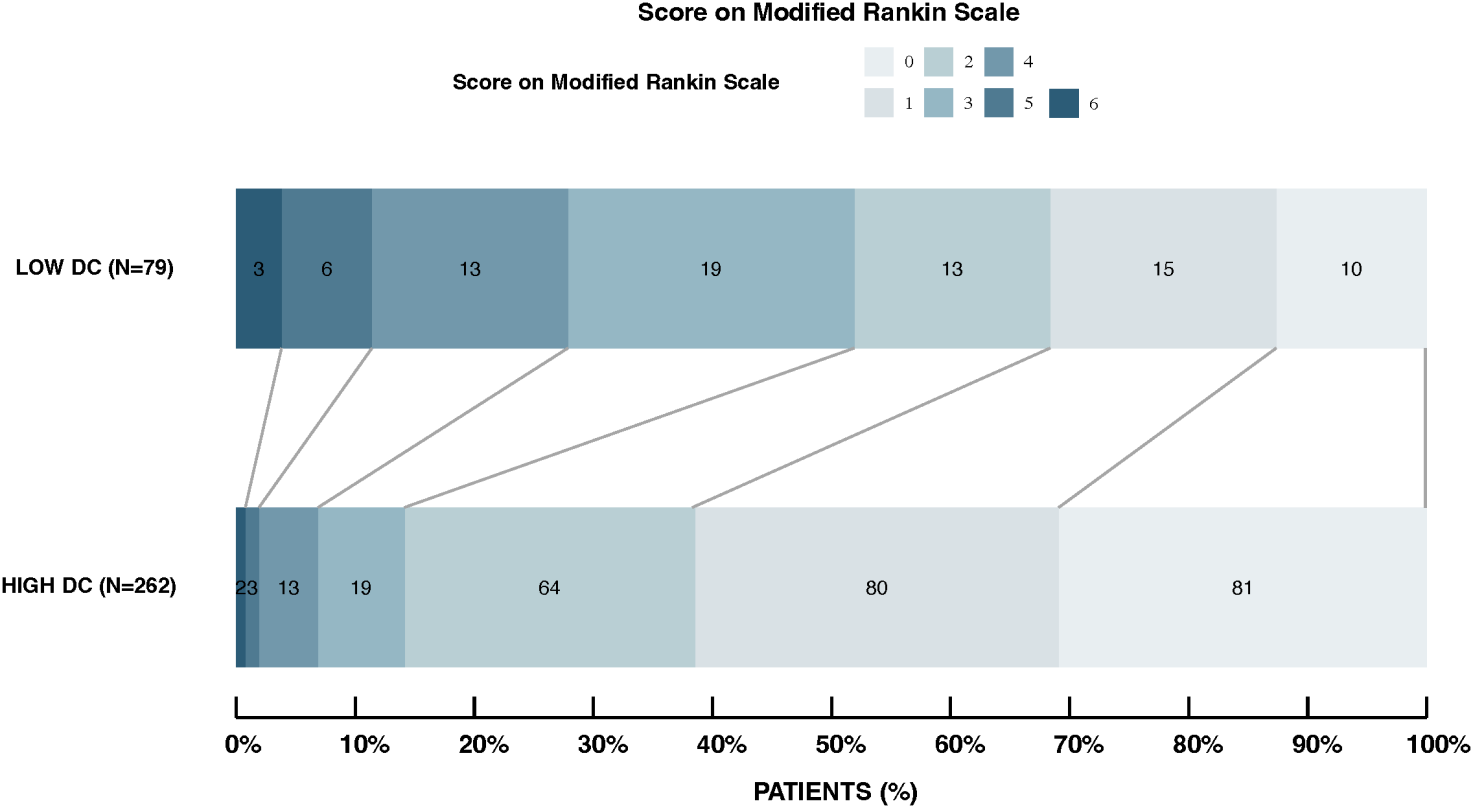
Distribution of modified rankin scale scores in high DC vs. low DC groups. This figure displays the 3-month post-stroke mRS score distributions for patients with high and low DC. The high DC group shows a higher proportion achieving favorable outcomes (mRS 0-2), while the low DC group has a more uniform spread across all mRS categories. Outcomes significantly differ between groups (p < 0.001).

The analysis of 3-month functional outcomes, shown in **Figure 4**, highlights a significant disparity between patients with high DC and low DC levels. In the high DC group (n= 262), a substantial proportion achieved favorable outcomes; 30.9% reached an mRS score of 0 (no symptoms) and more than 85% had mild or no disability (mRS 0-2). In contrast, the low DC group (n= 79) exhibited a more uniform distribution across the mRS spectrum, with only 12.7% achieving mRS 0 and a larger proportion were facing moderate to severe disability (mRS 3-6).

## 4 Discussion

This is the first study to investigate the relationship between DC and the prognosis of AIS. The results indicated that lower DC values (≤ 4.5 ms) during the acute phase were significantly associated with worse functional outcomes at 3 months after stroke, but not at 2 weeks, as measured by the mRS. An interesting aspect warranting further discussion is the potential for DC to predict longer-term outcomes and whether its predictive value evolves over time. In our study, although DC was not a significant predictor of outcomes at 2 weeks, it emerged as a robust predictor at 3 months, suggesting that its prognostic utility may become more apparent as the recovery process unfolds. This finding raises the possibility that DC, as a marker of autonomic function, may capture dynamic changes associated with the recovery trajectory in acute ischemic stroke patients. Future studies with extended follow-up periods—such as 6 months or 1 year—and serial DC assessments are needed to determine if the prognostic significance of baseline DC measurements is maintained, diminished, or enhanced over time. Furthermore, exploring whether interventions targeting autonomic function could modify these longer-term outcomes would be an important area for further research.

In our study, 24-hour Holter ECG monitoring was initiated within 48 hours of patient admission to capture early autonomic responses following AIS. However, it is important to consider that the post-stroke period is characterized by rapid and dynamic changes in autonomic function, influenced by the initial stress response, neuroendocrine alterations, and early therapeutic interventions. The timing of Holter monitoring may therefore play a critical role in the measurement of DC. For instance, recordings obtained within the first 24 hours might predominantly reflect acute vagal withdrawal and heightened sympathetic activation, whereas later measurements (e.g., beyond 48 hours) could capture a partial recovery of autonomic balance as the immediate stress subsides. This temporal variability suggests that serial assessments of DC at multiple time points post-stroke onset could offer a more nuanced understanding of autonomic dysfunction and its evolution during the recovery process. Future studies should investigate whether early versus later DC measurements yield differential prognostic information, which may ultimately refine the utility of DC as a biomarker for functional outcomes and guide the timing of potential interventions targeting autonomic regulation.

Patients with lower DC, which indicates poorer autonomic regulation, were found to have a higher prevalence of comorbidities, including hypertension and diabetes. The increased prevalence of diabetes and hypertension among patients with reduced DC may contribute to worse stroke outcomes by exacerbating autonomic dysfunction. Both diabetes and hypertension are known to impair cardiovascular autonomic regulation through mechanisms such as endothelial dysfunction, chronic inflammation, and altered neural feedback, which can lead to decreased vagal tone (20, 21). In this context, diminished DC may not only reflect impaired parasympathetic activity but also serve as an indicator of the cumulative deleterious effects of these comorbidities on autonomic balance. The combination of these risk factors could synergistically augment cerebrovascular vulnerability and impair recovery after acute ischemic stroke. These findings suggest that a comprehensive approach addressing both autonomic dysfunction and its underlying metabolic and vascular determinants might be critical for improving prognostic stratification and clinical outcomes.

Additionally, a non-linear negative correlation between DC and stroke outcomes was observed, with a significant inflection at a value of 4.5 ms, below which the likelihood of unfavorable outcomes sharply increased. These findings suggest that DC could serve as an important predictor of short-term recovery, emphasizing its potential as a biomarker for assessing prognosis and guiding early intervention strategies in AIS.

Our findings suggest that lower DC is associated with poorer prognostic outcomes. Few studies have investigated the role of DC in stroke. Years ago, our team reported a negative correlation between DC and stroke severity, as assessed by NIHSS, and speculated that DC could predict the prognosis of AIS (5). Consistent with previous study, we found that NIHSS score was higher in the DC≤ 4.5 ms group than in the DC > 4.5 ms group, althrough the difference was not statistically significant. In this study, we conducted a follow-up study in a larger cohort, and validated the predictive value of DC for AIS prognosis with 3-month follow-up, but not at 2-week.

This study is consistent with previous research about HRV and stoke prognosis, which showed that cardiac autonomic dysfunction was associated with poor outcomes after stroke (22). In their study, HRV indicators such as SDNN, SDANN were used to reflect the autonomic function. However, in our study, we did not find SDNN to be a significant predictor in the Logistic regression models. PRSA is a new arithmetic for evaluating cardiac autonomic function, as it can distinguish between sympathetic and parasympathetic function, reflecting by AC and DC, respectively (10). In contrast to other indicators such as SDNN and AC, DC was found to be more valuable for predicting outcomes in conditions like myocardial infarction (23, 24), heart failure (25), drug-induced cardiotoxicity (26), and non-ischemic dilated cardiomyopathy (27). This is the first study to explore the DC value in predicting prognosis of AIS. More studies are needed to compare the advantages of DC or HRV in the field.

Mechanistically, the observed relationship between DC and poor stroke outcomes can be explained by autonomic dysfunction patterns in stroke patients (28). Reduced DC directly reflects impaired vagal modulation, and this vagal dysfunction may have significant implications for stroke recovery through several pathways (29). Studies have shown that vagal nerve stimulation can enhance recovery by reducing neuroinflammation via the cholinergic anti-inflammatory reflex (30, 31). Experimental evidence from animal models has demonstrated that vagal nerve stimulation, when combined with rehabilitation, improves limb recovery following ischemic stroke (32). The prospective design of our study provides an important advantage in understanding the temporal relationship between autonomic dysfunction and stroke outcomes.

Furthermore, we employed several advanced analytical approaches to strengthen our findings. Based on research on cardiac diseases, we used a threshold of less than 4.5 ms for autonomic nerve function damage in patients with AIS. Coincidentally, we utilized RCS analysis to model the non-linear relationship between DC and stroke outcomes, revealing a critical threshold exactly at DC = 4.5 ms, below which the risk of unfavorable outcomes significantly increased. Our logistic regression models demonstrated that DC and age were robust predictors of stroke prognosis, with the 3-month model exhibiting superior discriminative ability (AUC = 0.744). Additionally, we conducted VIF analysis to address multicollinearity among HRV parameters (33), ensuring the stability and reliability of our predictive models. These methodological innovations provide valuable insights into the role of DC as an early prognostic marker in stroke recovery.

While our findings support DC as an early prognostic marker in AIS, a direct comparison with established prognostic markers could further elucidate its unique clinical value. Traditional markers such as the NIHSS score, imaging‐derived stroke volume, and various circulating biomarkers have been shown to robustly predict stroke outcomes. Evaluating the predictive performance of DC alongside these conventional measures may reveal whether DC offers complementary or even superior prognostic information. Future studies should focus on developing integrated prediction models that combine DC with these established indicators to enhance risk stratification and inform clinical decision‐making in AIS.

Several limitations of our study should be acknowledged. First, although this was a prospective study, it was conducted at a single center, which may limit the generalizability of our findings to broader populations with diverse demographic and geographic characteristics. Second, while we identified significant associations between DC and 3-month outcomes, we only measured DC at admission and did not perform follow-up measurements to assess potential changes in autonomic function during the recovery period. Third, although our statistical analysis addressed the multicollinearity among autonomic parameters, the sample size might not have been large enough to fully explore all potential confounding factors influencing the relationship between DC and stroke outcomes. Future research should focus on multicenter studies with larger sample size and serial DC measurements to validate our findings and gain a deeper understanding of the dynamic changes in autonomic function during stroke recovery.

## 5 Conclusion

In conclusion, this study highlights the potential of DC as a significant prognostic marker for predicting short-term outcomes in AIS patients. Our findings demonstrate that lower DC values are associated with poorer functional recovery, as measured by the mRS. This result emphasizes the role of autonomic dysfunction, specifically parasympathetic impairment, in the unfavorable prognosis of AIS. Future research should focus on validating these findings in larger, multicenter studies to improve the generalizability.

## Data Availability

The original contributions presented in the study are included in the article/**Supplementary Material**. Further inquiries can be directed to the corresponding authors.
