# Supplementary material for: Prognostic value of heart rate deceleration capacity for functional outcomes in acute ischemic stroke: a prospective study
Source: Front Endocrinol (Lausanne). 2025 May 13;16:1601346. doi: 10.3389/fendo.2025.1601346 (PMC12123432; doi:10.3389/fendo.2025.1601346)
Supplement: Supplementary file 1 [file DataSheet1.pdf]

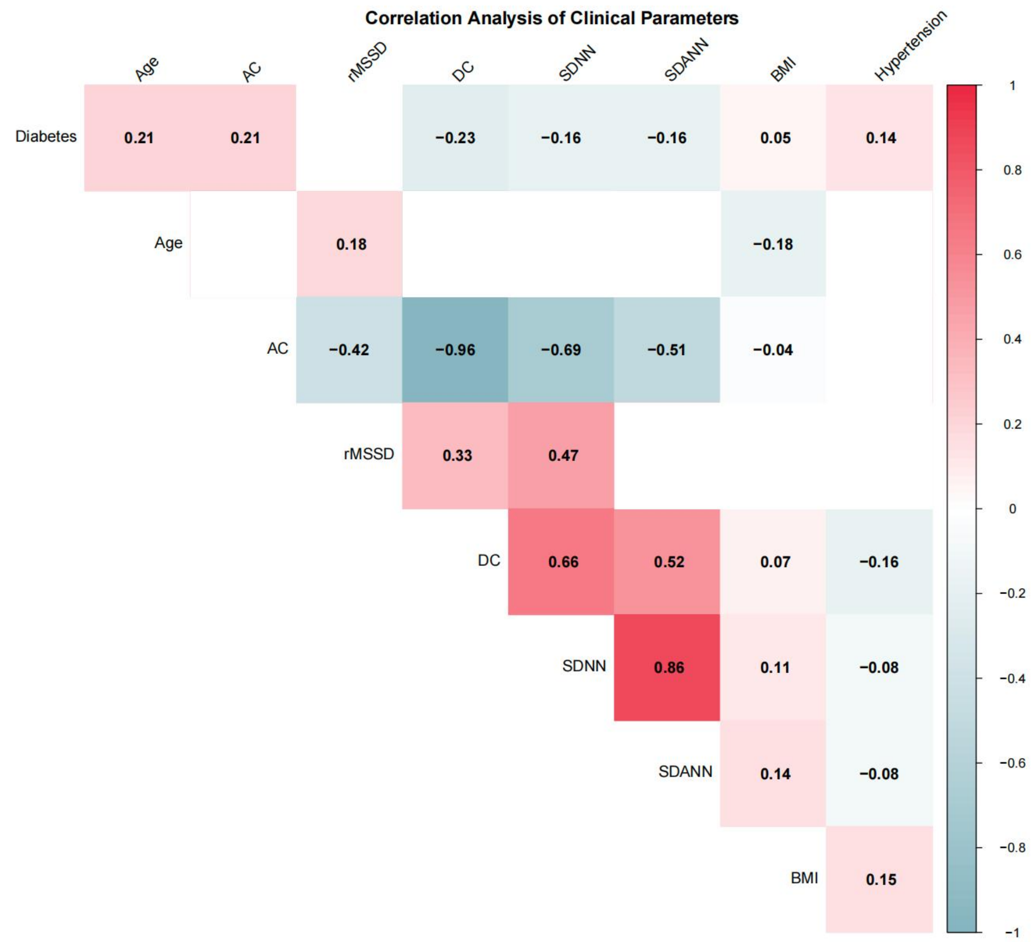

Supplementary Figure 1. Correlation Analysis of Clinical and Autonomic Parameters at Baseline. Correlation matrix showing Spearman's rank correlations between clinical parameters (age, BMI, diabetes, hypertension) and cardiac autonomic measures (AC, DC, SDNN, SDANN, rMSSD). The color intensity and size of the circles are proportional to the correlation coefficients. Red colors indicate positive correlations, while blue colors indicate negative correlations. Only statistically significant correlations ($P < 0.05$) are displayed. The correlation coefficients are shown in the upper triangle of the matrix. AC: acceleration capacity; DC: deceleration capacity; SDNN: standard deviation of normal-to-normal intervals; SDANN: standard deviation of the average normal-to-normal intervals; rMSSD: root mean square of successive differences; BMI: body mass index.
